# Protective effect of alamandine on doxorubicin‑induced nephrotoxicity in rats

**DOI:** 10.1186/s40360-021-00494-x

**Published:** 2021-05-29

**Authors:** Ava Soltani Hekmat, Ameneh Chenari, Hiva Alipanah, Kazem Javanmardi

**Affiliations:** Department of Physiology, Fasa University of Medical Sciences, Ebn-E-Sina SQ, Fasa, Iran

**Keywords:** Alamandine, Doxorubicin, Kidney, Cytokine, Oxidative Stress, Apoptosis, Immunohistochemistry

## Abstract

**Background:**

This study aimed to evaluate the protective effects of alamandine, a new member of the angiotensin family, against doxorubicin (DOX)-induced nephrotoxicity in rats.

**Methods:**

Rats were intraperitoneally injected with DOX (3.750 mg/kg/week) to reach a total cumulative dose of 15 mg/kg by day 35. Alamandine (50 µg/kg/day) was administered to the rats via mini-osmotic pumps for 42 days. At the end of the experiment, rats were placed in the metabolic cages for 24 h so that their water intake and urine output could be measured. After scarification, the rats’ serum and kidney tissues were collected, and biochemical, histopathological, and immunohistochemical studies were carried out.

**Results:**

DOX administration yielded increases in pro-inflammatory cytokines, including interleukin (IL)-1β and IL-6, pro-fibrotic proteins transforming growth factor-β (TGF-β), pro-inflammatory transcription factor nuclear kappa B (NF-κB), kidney malondialdehyde (MDA), creatinine clearance, blood urea nitrogen (BUN), and water intake. On the other hand, the DOX-treated group exhibited decreased renal superoxide dismutase (SOD), renal glutathione peroxidase (GPx) activity, and urinary output. Alamandine co-therapy decreased these effects, as confirmed by histopathology and immunohistochemical analysis.

**Conclusions:**

The results suggest that alamandine can prevent nephrotoxicity induced by DOX‎ in rats.

## Introduction

Doxorubicin (DOX) is an antibiotic anthracycline that was first isolated in 1960 and has been used for the past 30 years to treat cancer patients [1]. DOX has several side effects, such as cardiac and renal toxicity. DOX-mediated nephropathy is caused by glomeruli destruction and tubule damage [2, 3]. DOX-induced nephrotoxicity has been studied much less than other anthracycline-related side ‎effects. ‎DOX induces nephropathy via a complex mechanism. Free radicals, lipid peroxidation, and decreased antioxidant enzyme activity are among the most likely primary mediators in developing nephrotic syndrome [3, 4]. Inflammation also plays a significant role in DOX-induced kidney damage via the effects of topical cytokines and other cytotoxic factors [2]. DOX can also cause proteins to be excreted in the urine by destroying the nephrons directly.

DOX-induced nephropathy is a classic model of renal failure in rats. Damage to the filtration barrier is the primary cause of protein excretion due to renal failure [2]. DOX is responsible for this filtration barrier damage [5, 6].

Thus, administering drugs with anti-inflammatory and antioxidant effects could mitigate the side effects of DOX. Alamandine is a new member of the renin-angiotensin system (RAS) family that protects the cardiovascular system and kidney functions [7].

Alamandine, like angiotensin (1–7) [Ang (1–7)], is a heptapeptide. It differs from Ang (1–7) in only one amino acid in the N-terminal region. It exerts its effects by binding to the Mas-related G-protein coupled receptor of the type D (MrgD) receptor[8–10]. The renal actions of Ang (1–7 (might be related to reductions in oxidative stress and the activation of anti-apoptotic ‎pathways [11].

Lu et al. showed that Ang (1–7) protects kidneys from injuries caused by hypoxia by reducing oxidative ‎stress, fibrosis, and inflammation‎ [11]. Also, Mori et al. used a model of diabetic nephropathy to show that Ang‏ ‏‎(1–7‎‏ (‏can protect the kidneys by ‎reducing inflammation, oxidative stress, and lipotoxicity [12]. In addition, it was recently reported that ‎Ang (1–7) ‎ reduces DOX-induced cardiotoxicity in rats through antioxidant mechanisms[13].

Alamandine also has anti-fibrotic and anti-inflammatory effects [7, 14]. For example, alamandine increases antioxidant expression in ventricles exposed to ‎ischemia-reperfusion injury[15].‎Moreover, alamandine reduces pro-inflammatory factors induced by aortic ‎constriction, such as ‎TNF-α and IL-1α, in mice ‎ ‎[16]. In a study on an animal model of sepsis induction by polysaccharides in C57BL6/J mice, plasma and tissue levels of interleukin-1B (IL-1B) and tumor necrosis factor alpha (TNFα) increased. At the same time, alamandine reduced the levels of inflammatory cytokines and apoptosis in cardiac tissues [17]. To the best of our knowledge, no prior studies have examined the effects of alamandine on renal function and DOX-induced nephrotoxicity.

Many experimental and clinical reports have highlighted the protective role of Ang (1–7) in renal hemodynamics and functions under different conditions [18]. Also, alamandine has similar effects to Ang (1–7) [9, 19]. Therefore, we assumed that alamandine protects against DOX-induced nephrotoxicity.

In this study, several biomarkers of renal injury were measured in DOX-treated rats and alamandine/DOX co-administrated rats. These biomarkers included blood urea nitrogen (BUN)‎; creatinine; creatinine clearance; serum urea; albumin; and pro-inflammatory cytokines, including IL-1β and IL-6‎, pro-inflammatory transcription factor nuclear kappa B (NF-κB)‎, pro-fibrotic proteins transforming growth factor-β (TGF-β)‎, and renal antioxidant enzymes [superoxide dismutase (SOD), glutathione peroxidase (GPx), and malondialdehyde (MDA)‎]. Histological and immunohistochemistry analyses were also performed.

## Materials and methods

### Materials

This experiment was performed on 35 male Sprague-Dawley rats (180–220 g) obtained from the Experimental Animal Centre of Fasa University of Medical Sciences. All procedures followed relevant guidelines and regulations regarding the care and use of animals for the experimental procedures. The procedures were also approved by the Committee of Animal Care of the Fasa University of Medical Sciences (IR.FUMS.REC.1397.014) in compliance with the ARRIVE guidelines [20]. All animals were acclimatized under the controlled standard conditions of 12 h light/12 h dark cycles, at a temperature of 20–22 °C, standard pellet diets, and water ad libitum for one week before the start of experiments. Alamandine and DOX were obtained from Phoenix Pharmaceuticals Inc., CA, USA, and Tocris Bioscience, respectively.

### Experimental group design

After one week of acclimatization to cages, the rats were randomly divided into five groups:

1. The control group comprised intact animals that experienced no surgical interventions‎ and were given no drugs.
2. The sham group received normal saline as a vehicle for 42 days via mini-osmotic pumps (model 2006; ALZET Osmotic Pumps, CA, USA) that were surgically placed subcutaneously between the scapulae. This group also received normal saline intraperitoneal (i.p) on days 14, 21, 28, and 35.
3. The DOX group received DOX dissolved in normal saline (3.750 mg/kg) i.p on days 14, 21, 28, and 35 to reach the total cumulative dose (15 mg/kg).
4. The alamandine group received alamandine dissolved in normal saline for 42 days by mini-osmotic pumps with an infusion rate of 0.15 µl/h (50 µg alamandine/kg/day) as described by ‎Liu et al. [21].
5. The DOX + alamandine group received alamandine by mini-osmotic pumps for 42 days (50 µg alamandine/kg/day) and DOX (3.750 mg/kg) i.p on days 14, 21, 28, and 35 to reach the total cumulative dose (15 mg/kg) as described by Warp et al. [22].

On day 41, rats were housed in the individual metabolic cages. A 24-h urine sample was collected from each animal so that the creatinine clearance, creatinine, albumin, and TGF-B levels could be measured. On day 42 (seven days after the final DOX injection), the animals were euthanized with pentobarbital sodium (150 mg/kg, intraperitoneal, IP). Blood samples were taken, and both kidneys were removed from all rats. The blood samples were centrifuged at 4000 rpm for 10 min. Serums, which were used to evaluate biochemical parameters, were taken and maintained at -80 °C. Creatinine clearance (used to estimate the glomerular filtration rate (GFR)) was determined as described by Iyalomhe et al. [23] and Ogundipe et al. [24] This was done based on the 24-h urine samples obtained under the following formula (expressed as ml/min): Creatinine clearance (ml/min) = mg creatinine/dl urine×ml urine 24 h/mg creatinine/dl serum×1440.

Rats’ right kidneys were dissected and washed with PBS (10 mM PO43−, 137 mM NaCl, and 2.7 mM KCl; pH = 7.4). They were then dried on filter paper and weighed. Afterward, they were homogenized in the PBS and centrifuged at 10,000×g for 20 min at 4 °C. The supernatants were stored at -20 °C until the analyses of oxidative stress parameters, antioxidants, and TGF-β. These analyses were carried out using Eliza kits.

### Assessment of Water Intake and urine volume

Water intake and urine volume were assessed using metabolic cages. Water intake was calculated as the difference between the final volume measured and the remaining volume over 24 h. This measurement was considered as the daily water intake for every animal in each experimental group.

The volumes for both water intake and urine output were measured using measuring cylinders.

### Assessment of inflammatory cytokines in serum

The ‎ IL-6 ‎assay kit (cat. no. SEA079Ra), ‎NF-κB assay kit (cat. no. SEB824Ra)‎, and IL-1β assay ‎kit (cat. no. SEA563Ra)‎ were purchased ‎from Cloud-Clone Corp (Cloud-Clone Corp ‎Technology co., Ltd., Wuhan, China). Briefly, for IL-6 assay,‎100 µl of a standard blank or ‎samples was added into the ‎appropriate ‎wells and then incubated for 1 h at 37 °C. After ‎aspiration, 100 µl of prepared detection reagent ‎was added to each well and incubated for 1 h at ‎‎37 °C‎‏.‏‎ After aspiration and washing, 90 µL of ‎substrate solution was added to wells and incubated ‎for 15 min at 37 °C. Finally, 50 µL of stop ‎solution was added, and OD was ‎ immediately measured with a BioTek ELISA reader at 450 nm. ‎ The IL-6 concentration was ‎determined by comparing the OD of the samples to the standard curve.‎ The protocols for IL-‎1β‎ and NF-B assays were ‎similar to those used for IL-6.‎.

### Assessment of oxidative stress markers and TGF-β in kidney tissue and urine

TGF-β levels in the urine and kidney tissue were determined using ELISA kits (cat. no. DB100; R&D Systems, Inc., Minneapolis, MN), according to the manufacturer’s protocol. All measurements were performed in duplicate. Urine samples were collected in metabolic cages and centrifuged for 1 min at 10,000×g before being assayed or aliquoted and stored at -20 °C. As part of the assay procedure, 50 µl of assay diluent was added to each well. Afterward, 50 µl of standard, control, or sample was added to each well and incubated for 2 h at ‎‎room temperature. After aspiration and washing, 100 µl of a polyclonal antibody specific for TGF-β1 conjugated to horseradish peroxidase was added to each well and incubated for 2 h at ‎‎room temperature. After aspiration and washing, ‎100 µl of substrate solution was added to each well and incubated for 30 min at ‎‎room temperature.‎ Then, 100 L of a stop solution was added, and color changes were measured at 450 nm using a BioTek ELISA reader. ‎ The protocols for measuring TGF-β1 levels in kidney tissues were similar to those used for the urine samples.

‎The markers for oxidative stress, MDA, SOD, and GPx in kidney tissue homogenates were determined using available kits according to the manufacturer’s protocol. The MDA‎ assay kit ‎(cat. no. ZB‏-‏MDA‏-‏‎96 A‎)‎ and SOD assay kit ‎(cat. no. ZB‏-‏SOD‏-‏‎96 A)‎ were purchased from ZellBio (Ulm, Germany).

The thiobarbituric acid (TBA) method was used to determine MDA levels. The products of lipid hydroperoxide decomposition can condensate with TBA to form red compounds with an absorption peak at 532 nm. SOD measurements were based on the conversion of superoxide anion into hydrogen peroxide and oxygen. Absorbance at 420 nm was used to quantify SOD activity.

The GPx‎ assay kit ‎ (cat. no. RS 506)‎ was obtained from Randox Laboratories Limited, Crumlin, UK. The ‎oxidation of glutathione by cumene hydroperoxide was catalyzed by GPx. In the presence of NADPH and ‎glutathione reductase, oxidized glutathione was converted into a reduced form with a concomitant ‎conversion of NADPH to NADP+. The decrease in absorption was determined at ‎‎340nm using a spectrophotometer.‎.

### Immunohistochemistry (IHC)

IL-6, IL-1, P53, and NF-κB expressions were evaluated on paraffin-embedded tissues by a standard immunostaining assay. Briefly, xylene and a graded alcohol series were used for deparaffinization and rehydration, respectively. Then the slides were incubated for 30 min in a blocking reagent containing 1.5 % hydrogen peroxide in methanol. Antigens were retrieved and placed on slides using a microwave protocol. They were then incubated in serum for 30 min and immunostained with IL-6 (cat. no. sc-28,343; Santa Cruz Biotechnology, Inc.), IL-1 (cat. no. sc-32,294; Santa Cruz Biotechnology, Inc.), P53 (cat. no. sc-81,168; Santa Cruz Biotechnology, Inc.), and NF-κB (cat. no. sc-48,366; Santa Cruz Biotechnology, Inc.) primary antibodies for 1 h at room temperature.

Afterward, the slides were washed with PBS three times and then incubated with secondary antibodies for 30 min. The sections were stained using 3,30-diaminobenzidine (Dako liquid DAB color solution), and the slides were then counterstained with hematoxylin. An Olympus BX51 microscope (Olympus, Tokyo, Japan) was used to visualize the results. Five different microscopic fields were randomly selected from each slide, and positive staining within each slide was measured using Image-Pro Plus 6.1. Finally, quantitative analysis was performed in a blinded manner.

### Histopathological studies

The left kidneys of male rats were harvested and immediately fixed in 10 % buffered formalin phosphate (pH 7.4) for histological tests. The tissue samples were dehydrated by being passed through graded concentrations of alcohol, cleaned in xylene to remove alcohol, incorporated in paraffin, and allowed to harden. Subsequently, 5-µm sections of the paraffin blocks were prepared by microtome and left floating in the water bath. These floating kidney sections were then mounted onto microscopical slides, placed into a drying oven at 60 °C, and stained with Harris’ hematoxylin and 1 % eosin. Afterward, histological examinations were carried out using light microscopy. All sections were graded for tubular degeneration, tubular necrosis, and tubular cast formation. The ‎severity of these pathological lesions was assigned as follows: score 0 was considered to be normal; score ‎‎1 as ‎ mild‎; score 2 as moderate‎; score 3 as severe.‎.

### Data and statistical analyses

Prism software was used for the statistical analysis. The results are expressed as means ± ‎SD. ‎All data sets were first tested for ‎normality using the D’Agostino and Pearson omnibus and ‎Brown-Forsythe tests to ensure the data satisfied the criteria of normal distribution and homogeneity of variances, ‎respectively. A one-way ANOVA was used to analyze all datasets with a normal distribution, and ‎homogeneity‎ was evaluated using Tukey’s test for post-hoc comparisons. When unequal SDs were detected, Brown-‎Forsythe and Welch’s ANOVA tests were ‎applied, followed by Dunnett’s T3 multiple-comparison test. If the data were not normally distributed and for analysing the results of kidney damage, the Kruskal Wallis test with Dunn’s post-hoc test were performed. ‎‎P values less than 0.05 were ‎considered statistically significant.‎.

## Results

### Effect of different treatments on water intake, urine output, and renal toxicity markers

Water intake and urine output were checked one week after DOX injection (Table 1). Higher water intake (p = 0.003) and lower urine output (*p* < 0.001) were observed in rats in the DOX group than in the control group. Co-therapy with alamandine significantly decreased the effects of DOX. A comparison with the control group revealed that alamandine alone had a diuretic effect.

**Table 1:** Effect of treatment with alamandine on water intake, urine output and renal toxicity markers in DOX-treated rats

	Sham	Control	Ala	Dox	Dox + Ala
***Water Intake (ml/kg/day)***	63.10 ± 8.68	62.69 ± 6.90	70.31 ± 6.68	81.11 ± 9.84^** ††^	66.24 ± 10.21^#^
***Urine output*** ***(ml/kg/day)***	27.29 ± 3.72	27.57 ± 3.55	34.03 ± 4.13^###*†^	14.39 ± 4.34^***†††^	31.20 ± 4.90^###^
***Serum Creatinine (mg/dl)***	0.71 ± 0.19	0.65 ± 0.16	0.67 ± 0.23	1. 61 ± 0.31^***†††^	1.06 ± 0.38^##^
***Serum BUN*** ***(mg/dl)***	22.81 ± 4.99	22.98 ± 5.21	19.22 ± 6.33	43.45 ± 13.44^***†††^	28.32 ± 9.23^#^
***Serum Albumin (g/dl)***	4.06 ± 0.51	4.32 ± 0.58	3.96 ± 0.68	2.34 ± 0.78^**††^	3.20 ± 0.81
***Urine Creatinine*** ***(mg/dl)***	76.12 ± 7.32	76.76 ± 6.98	81.24 ± 11.45	58.32 ± 13.82^*†^	68.98 ± 14.34
***Urine Albumin*** ***(mg/dl)***	1.03 ± 0.21	0.7 ± 0.19	1.07 ± 0.24	2.89 ± 0.36***^†††^	2.05 ± 0.39^###‎***†††‎^
***Creatinine Clearance (ml/min)***	0.57 ± 0.14	0.64 ± 0.17	0.90 ± 0.53	0.08 ± 0.03^**‎†‎^	0.40 ± 0.18

Data are expressed as mean ± SD; *n* = 7 for each group

‎^†^*‎P* < 0.05, ‎^†‎‎†^‎*P* < 0. 01, ‎^†‎‎†‎‎†^‎*P* < 0.001 compared to the sham‎;‎

^*^P < 0.05, ^**^P < 0. 01, ^***^P < 0.001 compared to the control;

^#^*P* < 0.05, ^##^*P* < 0. 01, ^###^*P* < 0.001 compared to the DOX group

One week after the last dose of DOX was administered, serum albumin was significantly lower (*p* = 0.002) than in the control group, and urinary albumin was significantly higher (*p* < 0.001). These changes in urinary albumin were significantly ‎(*p* < 0.001)‎ reduced when alamandine was administered (50 mg/kg/day).

DOX administration increased serum BUN (p ‎<‎ 0.001) and serum creatinine (*p* < 0.001) while significantly decreasing urine creatinine (*p* = 0.033). Co-treatment with alamandine significantly reduced serum BUN (*p* = 0.017) and serum creatinine (*p* = 0.005) compared to the DOX treatment. ‎Alamandine co-therapy produced an insignificant increase in urine creatinine (*p* = 0.405).

The results also revealed that creatinine clearance levels were lower in the DOX group than in the control group (*p* = 0.003). Rats co-treated with alamandine exhibited an insignificant increase in creatinine clearance compared to the DOX group ‎(*p* = 0.535).

## Effect of different treatments on inflammatory cytokines and NF-kB

DOX administration increased the serum levels of IL-1β (*p* = 0.005), IL-6 (*p* < 0.001) and NF-κB (*p* = 0.011). Alamandine co-therapy resulted in lower IL-6‎ levels when compared to therapy using DOX alone ‎(*p* = 0.040). Insignificant reductions were observed in IL-1β and NF-B levels ‎(*p* = 0.439 and *p *= 0.091, respectively)‎.

TGF-β levels in the kidney and urine were higher in the DOX group than in the control group (*p* < 0.001). Co-treatment with alamandine decreased these levels ‎(p = 0.009 for the kidney and p = 0.002 for the urine) (Table 2).

**Table 2:** Effect of treatment with alamandine on inflammatory cytokines and NF-kB in DOX-treated rats

	Sham	Control	Ala	Dox	Dox + Ala
***TGF-β in Urine*** ***(pg/mg creatinine)***	16.48 ± 5.69	17.20 ± 5.50	23.34 ± 8.44	79.89 ± 15.78^***†††^	56.22 ± 13.33^***##†††^
***TGF-β in Kidney*** ***(pg/mg protein)***	142.7 ± 45.99	135.9 ± 22.56	179.1 ± 67.20	534.4 ± 99.90^***†††^	404.4 ± 77.23^***##†††^
***Serum IL-1훽 (pg/ml)***	363.4 ± 99.9	350.0 ± 86.8	342.2 ± 89.99	552.1 ± 109.3^**††^	453.9 ± 105.3
***Serum IL-6 (pg/ml)***	22.32 ± 5.53	20.10 ± 5.34	21.23 ± 6.56	47.46 ± 10.32^***†††^	34.98 ± 9.77^**#††^
***Serum NF-kB (ng/ml)***	6.5‎±‎2.1	6.3‎±‎1.9	6.4‎±‎1.9	10.2‎±‎2.2*^†^	7.3‎±‎2.1

Data are expressed as mean ± SD; *n* = 7 for each group

‎^†^‎ *P* < 0.05, ‎^†‎‎†^‎*P* < 0. 01, ‎^†‎‎†‎‎†^‎*P* < 0.001 compared to the sham‎;‎

‎^*^P < 0. 05, ‎^**^P < 0. 01, ^***^P < 0.001 compared to the control;

^#^*P* < 0.05, ^##^*P* < 0. 01 compared to the DOX group

### Effects of different treatments on renal oxidative stress markers

DOX administration markedly increased kidney MDA (*p* = 0.005). The reduction induced by alamandine Co-therapy was not statistically significant‎ (*p* = 0.999)‎. DOX administration caused a significant decrease in kidney SOD (*p* = 0.006) and a significant decrease in renal GPx activity (*p* = 0.004) relative to rats in the control group. Alamandine co-therapy insignificantly diminished these differences‎ (*p* = 0.554 for kidney SOD and *p* = 0.999 for GPx activity)‎ (Table 3).

**Table 3:** Effect of treatment with alamandine renal oxidative stress markers in DOX-treated rats

	Sham	Control	Ala	Dox	Dox + Ala
***Kidney SOD (U/g tissue)***	71.90 ± 10.62	74.42 ± 9.62	69.51 ± 11.96	48.52 ± 14.93^**†^	59.04 ± 16.09
***Kidney MDA (nmol/g tissue)***	4.44 ± 1.53	4.46 ± 0.94	5.52 ± 2.61	17.3 ± 5.31^**††^	14.87 ± 5.36^*†^
***Kidney GPx Activity*** ***(u/mg protein)***	11.17 ± 2.81	11.72 ± 2.09	10.26 ± 2.51	4.91 ± 2.89^**†^	6.61 ± 2.88

Data are expressed as mean ± SD;* n* = 7 for each group

‎^†^‎ *P* < 0.05, ‎^†‎‎†^‎*P* < 0. 01 compared to the sham‎;‎

‎^*^P < 0. 05, ‎^**^P < 0. 01, ^***^P < 0.001 compared to the control

### Immunohistological study

**Fig. 1:**
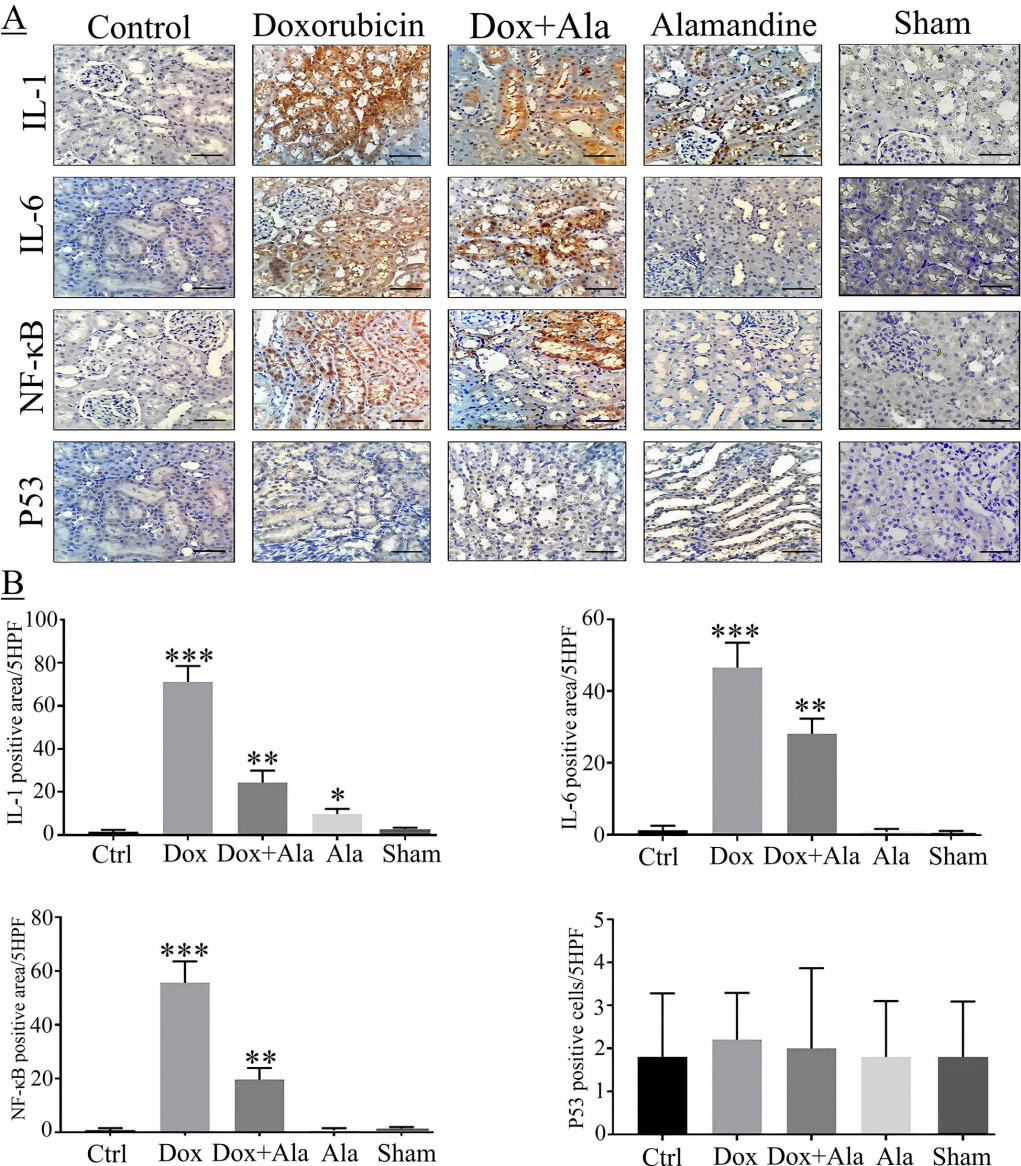
**a**: Immunohistochemistry of IL-1, IL-6, P53, and NF-Κb in the kidney, scale bar 100 μm. Magnification ×400. **b**: Immunohistochemical analysis of IL-1, IL-6, P53, and NF-Κb in the kidney. *, **, ***: values indicate treatment group versus control group; ***p < 0.001, **p < 0.01, *p < 0.05

All immunohistochemical sections of kidney tissues are presented in Fig. 1. The minimum expressions of IL-6, IL-1, P53, and NF-κb were seen in the control group. Moreover, the expression levels of P53 were negligible in all groups. Intense staining of IL-6, IL-1, and NF-κb was observed in the DOX group (p < 0.01).

However, the expression of pro-inflammatory cytokines (IL-1, IL-6, and NF-κb) was lower in the alamandine + DOX treatment group than in the DOX group. The results obtained from the alamandine group were similar to those of the control group, except for the finding that IL-1 expression was significantly higher in the alamandine group (Fig. 1).

### Histopathological study

**Fig. 2:**
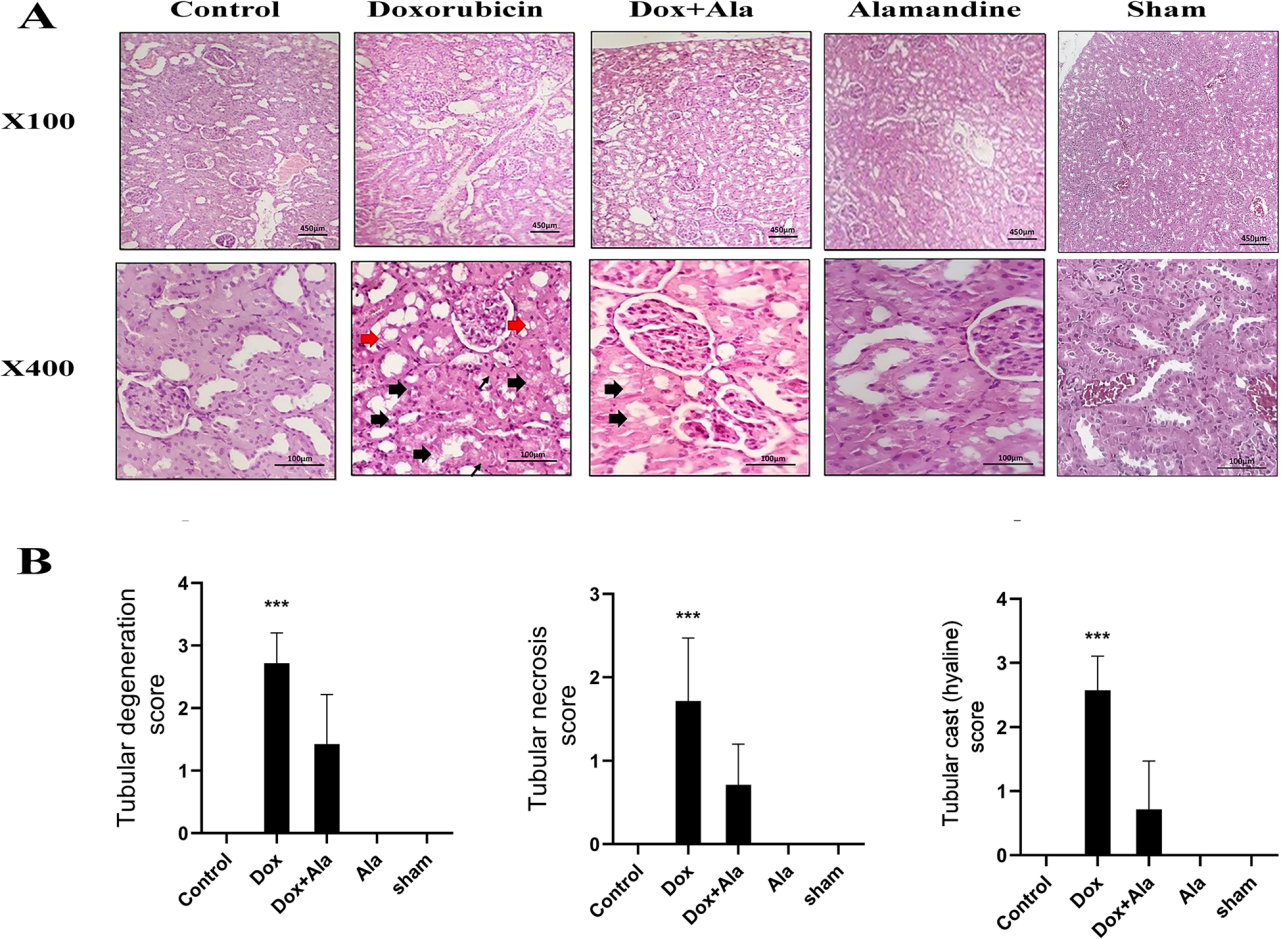
**a** Histopathologic sections of kidneys from different experimental groups. thick black arrows = degeneration of tubular cells; thin arrows = cell necrosis; red arrows = hyalin cast. H&E stain. **b** The histopathological score analysis of kidney tissue damage demonstrates the effect of DOX with and without alamandine. A score of 0 indicates normal, a score of 1 indicates mild, a score of 2 indicates moderate, and a score of 3 indicates severe.^***^P < 0.001 versus respective control

All hematoxylin and eosin (H&E) stained kidney sections from different experimental groups were evaluated histologically. The kidney sections of the alamandine group did not show any significant histopathological changes However, the histopathological evaluation of the kidneys of DOX-treated animals showed severe ‎tubular degeneration, tubular cast and moderate tubular necrosis‎. Alamandine co-therapy ‎decreased these pathological changes‎ (Fig. 2).

## Discussion

DOX is used to treat multiple solid tumors but has severe adverse effects on the kidneys [25, 26]. The mechanisms by which DOX causes glomerular toxicity have are not yet fully understood. However, previous reports have shown that reactive oxygen species and free radicals are the most significant contributors to DOX-induced nephrotoxicity [26–28]. The conversion of DOX to its semiquinone is thought to play an essential role in its nephrotoxic actions[29]. Semiquinone is unstable under aerobic conditions and, therefore, reacts with molecular oxygen to form superoxide anion radicals[30]. As the number of primary free radicals increases, locally infiltrating neutrophils and active mesenchymal glomerular cells generate more free radicals that damage kidney tissues [31]. DOX exerts direct toxic damage to the glomerular base membrane, podocytes, and glomerular endothelial cells, inducing tubular interstitial inflammation and fibrosis[32]. As a result, renal function is compromised following DOX administration—in turn, serum urea and creatinine concentrations increase while serum albumin, urea, and creatinine clearance decrease, ultimately leading to extreme proteinuria [33, 34].

In the current research, we used a cumulative dose of DOX injections to induce an experimental nephrotic syndrome model. This model was characterized by albuminuria, hypoalbuminemia, increased serum levels of BUN and creatinine (two significant indicators of renal function), and decreased creatinine clearance) indicator of GFR), all of which are associated with increased oxidative stress and inflammatory factors.

The toxic effects of DOX administration observed in the current study are consistent with the findings of previous studies in which renal function parameters, including serum urea and creatinine, increased [35, 36]. However, administering almandine before, during, and after DOX injections yielded improvements in renal function parameters, including attenuated serum urea, creatinine, albumin, and creatinine clearance. These improvements in DOX-induced renal dysfunction have been confirmed by histological and biochemical findings. For example, DOX has been shown to increase oxidative stress in the kidneys, as indicated by increased lipid peroxidation and changes in antioxidants’ status [37].

Oxidative stress is crucial to the development of podocyte damage, glomerular sclerosis, and proteinuria [38]. Our results showed that alamandine co-therapy restored the levels of ROS end products (MDA) and ROS-preventing enzymes (SOD and GPx) in the kidneys compared to the control group. Thus, this treatment could relieve renal oxidative stress in rats with DOX-induced nephrotoxicity. Alamandine’s protective effects on albuminuria and glomerular basement membrane damage could be partly explained by its antioxidant property. MDA can indicate lipid peroxidation, and it is used indirectly to assess how much damage has been done to cell membranes [39]. SOD catalyzes the dismutation reaction of superoxide anion to hydrogen peroxide, which is then detoxified into oxygen and water by catalase or glutathione peroxidase. Such proteins prevent damage caused by oxidative stress [40]. The results presented in this research are consistent with those of a previous study indicating that alamandine increased antioxidant protein expression in ventricles exposed to I/R injuries [41].

NF-kB is a transcription factor that regulates a wide variety of genes involved in developing renal disease [42, 43]. NF-kB activation plays a pivotal role in the pathogenesis of DOX-induced renal inflammation [44]. NF-kB is responsible for inflammatory reactions via the mediation of TNF-α, IL-1β, and IL-6 expressions [45, 46]. In this study, serum and tissue elevations of TNF-α, IL-1β, IL-6, and NFκB were observed in DOX-treated rats. Alamandine co-therapy reduced the extent to which NFκB, IL-1β, TNF-α, and IL-6 levels were altered by DOX. These results suggest that alamandine has anti-inflammatory effects and can reduce the harm done by DOX in harvested tissues.

In this study, alamandine alone increased IL-1. Macrophages have MrgD receptors [47], and it is has been proposed that alamandine binds these receptors, thus increasing IL-1 secretion [47]. However, this increase in IL production requires further investigation.

TGF-β was found to be a central mediator of renal fibrosis [48, 49]. TGF-β_1_ can be synthesized by a wide variety of cells, including macrophages, T and B fibroblast lymphocytes, and resident renal cells [50]. In the present study, alamandine reduced renal TGF-β_1_ levels. TGF-β_1_ expression in the kidneys is considered to be the final common pathway leading to structural damage and fibrosis in various glomerular diseases [51, 52]. Urinary TGF-β1 appears to be a marker of glomerular damage severity. In our study, urinary levels of TGF-β_1_ were also increased (and subsequently reduced by alamandine co-therapy).

P53 induction mediates cell apoptosis by activating the caspase-3 protease family and inducing apoptotic cell death [53]. However, in the present study, the expression of P53 IHC did not change in either the DOX or alamandine groups, indicating that DOX side effects are reduced independent of P53. Perhaps other pathways, including ROS, inflammatory cytokines, and NFκB, work together to damage the kidneys. The elimination of intracellular H_2_O_2_ protects myocytes from DOX-induced apoptosis, probably by inhibiting NF-κB activation [54]). Thus, alamandine could protect the kidneys by reducing superoxide and blocking the NF-B pathway.

The beneficial effects of alamandine were further illustrated by a histological evaluation using H&E staining. Histopathologic examinations showed marked pathological lesions. These lesions were characterized by severe proximal and distal tubular cell swelling (cell degeneration), tubular cell necrosis, and a deterioration of the architecture of the kidney. Alamandine treatment reduced these pathological lesions.

**Fig. 3:**
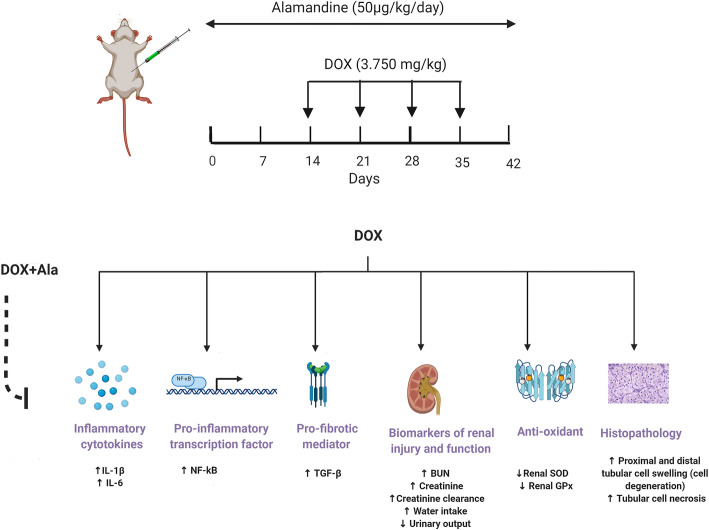
Effects of alamandine on DOX-induced nephrotoxicity

Surprisingly, the results of this study showed that alamandine alone increased urine volume. Evidence indicates that Ang (1–7) can counterbalance the vascular and tubular action of AngII [55, 56]. Ang (1–7) induces vasodilator, natriuretic, and diuretic effects through the mas receptor [57]. It has been suggested that alamandine, a heptapeptide with an Ang (1–7)-like structure, exhibits actions similar to Ang (1–7) [7, 58–60]. However, alamandine’s effects on urine volume require further investigation. Figure 3 is an overview of alamandine’s effects on DOX-induced nephrotoxicity.

Both alamandine and Ang-(1–7)‎, owing to their respective receptors Mas and MrgD, are members of the RAS protective arm. This counter-regulatory RAS axis has anti-oxidative, anti-inflammatory, and anti-fibrotic effects. The ACE2/Ang-(1–7)/Mas axis plays a protective role in some renal disease models. ‎A few human studies have revealed the beneficial effect of Ang-(1–7) [61]. However, further studies must be done to elucidate the downstream events triggered by alamandine/MrgD receptors that modulate this protective effect. Understanding the alamandine/MrgD receptor pathway can lead to new pharmacological strategies for repairing the pro-inflammatory environment that causes various diseases. Current research suggests that drugs that activate this protective arm of the RAS could improve treatments for various disorders, including renal diseases.‎.

Our results suggest that alamandine alleviates DOX-induced nephrotic syndrome in rats, likely owing to its antioxidant and anti-inflammatory properties. These findings indicate that alamandine could be used as a therapeutic agent for DOX-induced nephrotoxicity.

## Study limitations

Our study had several limitations, including the use of pentobarbital at such a high dose (150 mg/kg) during exsanguination. This dose ‎might have influenced some cytokines and biochemical parameters, such as liver ‎enzymes, urea ‎, and creatinine [62].

Another limitation is that the animals used in this study did not have cancer. Therefore, they did not exhibit all the cellular and molecular changes associated with ‎cancer pathophysiology.‎.

Nevertheless, understanding the mechanisms of DOX-induced nephrotoxicity at an early stage of the ‎disease is important for preventing additional health problems, such as nephrotoxicity, and resulting complications in cancer patients.

## Data Availability

The datasets used and/or analyzed during the current study are available from the corresponding author on reasonable request.
